# Endoscopic biliary drainage as a bridging procedure to single-stage surgery for perforated choledochal cyst: a case report and review of the literature

**DOI:** 10.1186/s40792-015-0115-4

**Published:** 2015-11-17

**Authors:** Takuya Minagawa, Shoichi Dowaki, Hiroyuki Kikunaga, Koji Fujita, Keiichi Ishikawa, Katsuaki Mori, Tadayuki Sakuragawa, Shunsuke Ichisaka, Hiroshi Miura, Koichiro Kumai, Shuji Mikami, Yuko Kitagawa

**Affiliations:** Department of Surgery, Hino Municipal Hospital, 4-3-1 Tamadaira, Hino, Tokyo 191-0062 Japan; Department of Radiology, Hino Municipal Hospital, 4-3-1 Tamadaira, Hino, Tokyo 191-0062 Japan; Division of Diagnostic Pathology, Keio University Hospital, 35 Shinanomachi, Shinjuku-ku, Tokyo 160-8582 Japan; Department of Surgery, Keio University School of Medicine, 35 Shinanomachi, Shinjuku-ku, Tokyo 160-8582 Japan

**Keywords:** Choledochal cyst, Perforation, Endoscopic biliary drainage, Adult, Single-stage surgery

## Abstract

Choledochal cyst (CC)—a congenital anomaly of the bile duct—is rare. We report a 28-year-old woman complaining of epigastralgia who was transferred to our hospital. Physical examination revealed severe tenderness to abdominal palpation without symptoms of diffuse peritonitis. Urgent contrast-enhanced abdominal computed tomography indicated the dilated common bile duct (CBD) was perforated, with a presumed diagnosis of perforated CC. Endoscopic external biliary drainage was performed immediately as a bridging procedure to the definitive surgery. Additional evaluations confirmed a type IVa CC, according to Todani’s classification, but no signs of malignancy. Twenty-two days after biliary drainage, laparotomy was performed. A large cystic mass was found in the CBD with a perforated scar on the right-side wall. Because inflammation around the pancreas head was too severe to perform cyst excision safely, the patient underwent subtotal stomach-preserving pancreatoduodenectomy. The postoperative course was uneventful, and the patient was discharged on the 29th postoperative day. Pathologic examination of a specimen showed no malignancy, and the patient has remained well during the 3-year follow-up. Our experience with this case suggests that definitive single-stage surgery for perforated CC in an adult can be performed safely owing to external biliary drainage as a bridging procedure, if manifestation of diffuse peritonitis is not evident.

## Background

Choledochal cyst (CC) in adults is rare [1], but perforation of CC occasionally has been reported following invasive procedures in or around the common bile duct (CBD) or after trauma [2–4]. Spontaneous perforation of CC is an extremely rare cause of abdominal pain in adults. Patients commonly undergo two-stage surgery comprising urgent peritoneal lavage and T-tube biliary drainage by laparotomy as the first surgery, and cyst excision and biliary reconstruction as the second surgery [5–9]. However, we believe there is a less invasive alternative to laparotomy for biliary drainage in many cases. Because more than 90 % of CCs are associated with anomalous pancreaticobiliary ductal union (APBDU) [10, 11], and because biliary ductal carcinoma occasionally is seen in adults with CC [12–14], it is essential to evaluate the bile duct thoroughly before performing the definitive surgery. Here, we report a thought-provoking case of spontaneous perforation of CC in an adult woman. The patient was successfully managed by endoscopic external biliary drainage followed by complete cyst excision. We also review past cases in the literature and discuss the problems associated with management and timing of the definitive surgery for perforated CC in adults.

## Case presentation

A 28-year-old woman experienced sudden-onset abdominal pain with associated nausea and vomiting during the night. She was transferred to our hospital complaining of severe epigastralgia. The patient’s medical history was unremarkable. Her blood pressure was 104/66 mmHg, her heart rate was 107 beats/min, and her body temperature was 37.7 °C. On physical examination, she had severe tenderness to palpation around the umbilicus with no signs of diffuse peritonitis. Laboratory results were as follows: white blood cell count, 13.3 × 10^9^/L; hemoglobin, 14.6 g/dL; aspartate aminotransaminase, 623 IU/L; alanine aminotransaminase, 431 IU/L; total bilirubin, 4.9 mg/dL; alkaline phosphatase, 1007 IU/L; gamma-glutamyl transpeptidase, 939 IU/L; and C-reactive protein, 3.7 mg/dL.

**Fig. 1:**
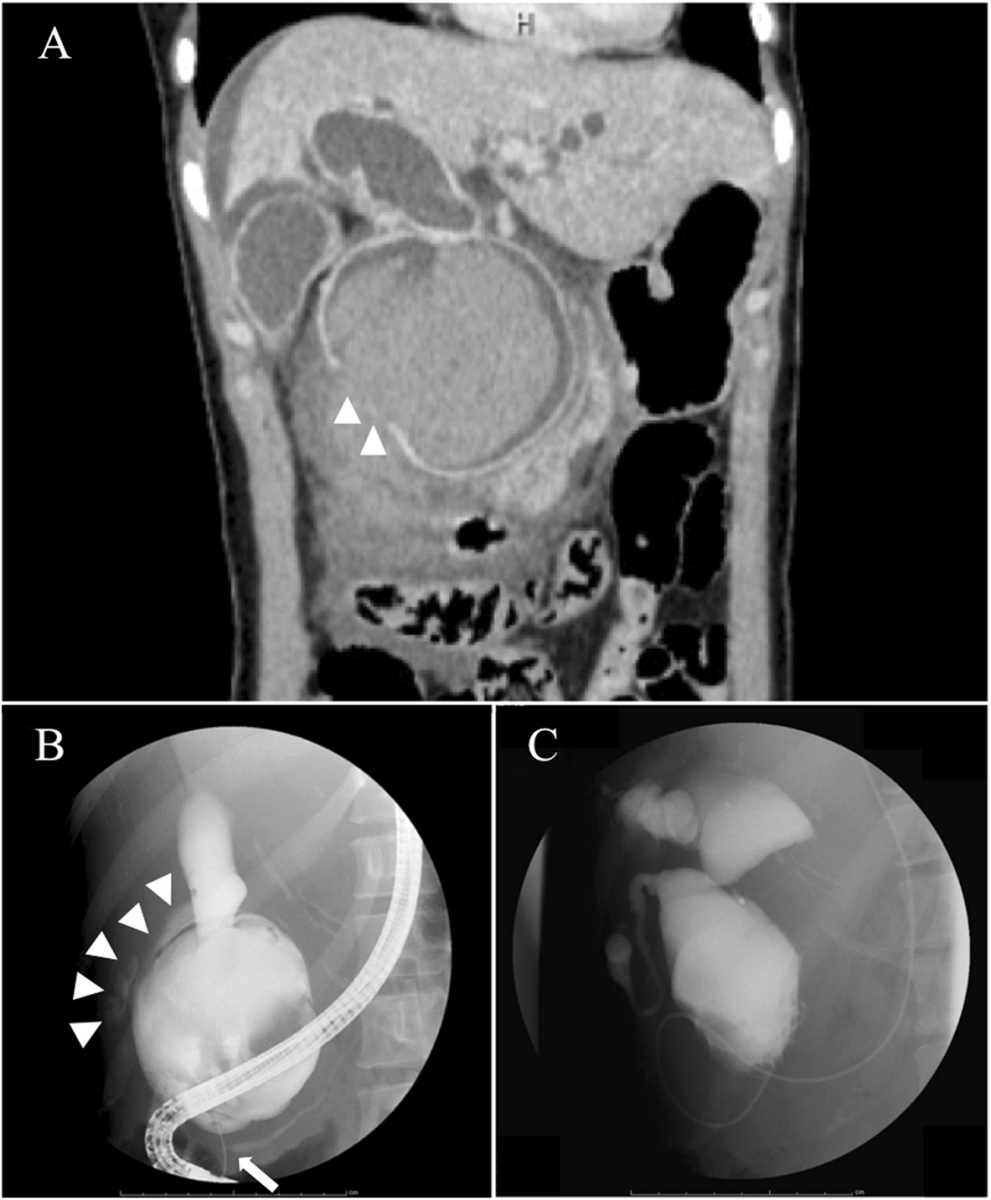
Preoperative imaging findings. **a** Urgent contrast-enhanced abdominal computed tomography scan, showing a huge cyst with hemorrhaging in the common bile duct (CBD) and a defect of the contrast medium at the right-side wall of the CBD (*arrowheads*). Fluid collection around the CBD, mainly in the retroperitoneal space, also is observed. **b** Endoscopic retrograde cholangiopancreatogram, showing leakage of the contrast medium from the right-side wall of the dilated CBD (*arrowheads*) with anomalous pancreaticobiliary ductal union (*arrows*). **c** Cholangiogram taken by the nasal biliary drainage tube, showing dilation of both intra- and extrahepatic bile ducts, indicating a type IVa choledochal cyst according to Todani’s classification

Urgent contrast-enhanced abdominal computed tomography (CT) revealed remarkable dilatation of the CBD. It also demonstrated fluid collection around the CBD, mainly in the retroperitoneal space, and suspicion of a perforated cyst wall (Fig. 1a). Endoscopic retrograde cholangiopancreatography (ERCP) was performed for further evaluation and biliary drainage, with a presumed diagnosis of perforated CC. ERCP revealed APBDU and biliary leakage out of the CC (Fig. 1b). The concentration of amylase in the bile was 2040 IU/L. After an endoscopic nasal biliary drainage (ENBD) tube was placed, the general condition of the patient improved. Before the definitive surgery, additional multimodal examinations, including cholangiography taken by the ENBD tube (Fig. 1c), CT, magnetic resonance cholangiopancreatography, ultrasonography, and pathology tests, revealed a congenital type IVa CC, according to Todani’s classification [6], but no signs of malignancy.

Twenty-two days after endoscopic biliary drainage, laparotomy was performed. Operative findings showed the bile duct was extremely dilated from the porta hepatis to the lower part of the CBD. A single free perforation site was observed at the right-side wall of the CBD, near the confluence of the cystic duct. The other organs had no evidence of perforation. Because inflammation around the pancreas head was severe, it was difficult to dissect the dilated intrapancreatic bile duct safely. For complete resection of the cystic mass, subtotal stomach-preserving pancreatoduodenectomy was performed, and the Traverso technique was employed for reconstruction. The postoperative course was uneventful, and the patient was discharged on the 29th postoperative day. Pathologic examination of a specimen revealed no malignancy, and the patient has remained well during the 3-year follow-up.

### Discussion

CC, or bile duct cyst—a congenital dilation of the biliary tract—is commonly seen in women, East Asians, and children. The incidence of CC in Western populations ranges from 1 in 100,000 to 1 in 150,000 live births [15], which is thought to be lower than that in Asian countries. In Japan, the total number of admissions to children’s hospitals for CC has been estimated to be as high as 1 in 1000 [16], although, it has been reported to be 1 in 13,500 in the USA [17] and 1 in 15,000 in Australia [18]. Biliary cysts can be divided into five subgroups according to Todani’s modification of the Alonso-Lej classification [19]. It has been reported that APBDU, which is seen in almost all cases of CC [10, 11], predisposes the patient to risk of carcinoma. Incidence of carcinogenesis associated with CC ranges from 4 % to 30 %, and increases with age [13, 14, 20–23]. Given the high risk of malignant conversion, surgical resection of the cyst, especially the extrahepatic bile duct, has been recommended for type IV CCs [23, 24].

Perforation of CC is a rare, but important, condition in children [25, 26]. Spontaneous perforation of CC in adults is extremely rare [6, 7]. Surgical management, especially two-stage biliary reconstruction, commonly has been performed for bile peritonitis [5–9]. There are several reasons why two-stage surgery has been strongly recommended. First, the cyst wall is so friable due to severe inflammation that anastomosis in primary closure would be disrupted. This is why T-tube drainage is recommended in the first surgery [27]. Second, because of bile staining and severe inflammation, a definitive procedure can be difficult to perform during emergency exploratory laparotomy. Therefore, it is safer to perform the definitive surgery when the patient recovers from the inflammatory state.

However, in adult cases with no signs of diffuse peritonitis, it might be possible to perform transient biliary drainage using an endoscopic or percutaneous approach. This strategy is not common, but it has several advantages. First, this strategy can be used as a less invasive alternative to emergency drainage by laparotomy for patients in bad general condition. Using endoscopic or percutaneous biliary drainage as a bridging procedure, definitive single-stage surgery can be performed when inflammation has improved and the patient is in better condition. The second advantage is that additional examinations can be performed before the definitive surgery while the patient recovers from the inflammatory state. These examinations could make the preoperative evaluation and diagnosis more precise, including assessment of malignant potential. Endoscopic or intraductal ultrasonography, for example, might be effective for preoperative diagnosis due to their high diagnostic accuracy [28, 29]. Bile cytology also could be helpful for diagnosis. Cholangiography taken by the cyst drainage tube would be extremely helpful to plan the optimal definitive surgery. If any signs of malignancy are indicated, R0 surgery and additional lymphadenectomy should be considered. In our case, owing to biliary drainage, multimodal examinations enabled precise evaluation, and helped to simulate the optimal definitive surgery.

There are some considerations to bear for this strategy. In our case, there were no evident indicators of histologic improvement from the inflammatory state. Rapid recovery from the acute phase and sufficient biliary drainage for almost 3 weeks allowed us to suspect improvement from the inflammatory state. However, the definitive surgery was complicated by residual inflammation around the pancreas head. Therefore, it can be difficult to determine proper timing of the definitive surgery following transient biliary drainage. It is easy to understand that biomarkers of inflammation, such as white blood cell count or serum C-reactive protein level, and patients’ symptoms indicate clinical improvement from the inflammatory state. However, these criteria do not always indicate histological improvement. As in the management of other inflammatory diseases, a sufficient interval might be needed for safe operation. An interval appendectomy for acute appendicitis in children is generally planned 2 or 3 months after conservative management [30]. Histological findings suggest that the adhesion around the appendix improves about 2 months after the disappearance of symptoms [31]. On the other hand, early cholecystectomy following biliary drainage for acute cholecystitis is reported to be preferable if the patient’s condition improves, because surgery is easy to perform when the inflammation has improved and the adhesion is still soft and fresh [32, 33]. Based on these findings, with regard to histological tissue inflammation, it might be better to plan elective surgery either within 72 h or over 2 months after the onset of symptoms. However, malignancy might be difficult to rule out within 72 h after the onset of symptoms. Therefore, in clinical practice, it might be recommended to perform elective surgery over 2 months after the onset and disappearance of symptoms if biliary drainage is carefully managed. Our patient required continuous biliary drainage due to extreme narrowing of the lower biliary tract; therefore, ENBD was thought to be a better intervention for biliary drainage than endoscopic biliary stenting (EBS). This is because ENBD can detect nonfunctioning of the biliary drainage system much earlier and because exchanging an ENBD tube for an EBS tube might be a challenging procedure with a high risk of re-perforation in this case. Our patient could not wait more than 2 months for elective surgery because of social issues; therefore, she underwent definitive surgery 3 weeks after the onset of symptoms.

If malignancy is suspected, endoscopic biliary drainage might be performed using a percutaneous approach with regard to peritoneal dissemination. Both endoscopic and percutaneous approaches are appropriate for biliary drainage near the CBD. On the other hand, it would be better to employ a percutaneous approach when bile collection is distant from the bile duct. If necessary, a combination of these approaches can be considered for better management.

In a PubMed database search, we found 10 cases in eight English articles addressing management of spontaneously perforated CC in adults [1, 5–9, 34, 35]. We summarize the features and clinical course of these 10 cases in Table 1. In most of these cases, patients were aged in their twenties, from East or South Asian countries, and presented with bile peritonitis. All 10 cases were either a type I or IVa CC, according to Todani’s classification, and had no signs of malignancy. Preoperative abdominal paracentesis confirmed biliary leakage in six cases. Two-stage surgery comprising T-tube choledochocystostomy and peritoneal lavage as the first surgery, and cyst excision and hepaticojejunostomy as the second surgery was performed in six cases. Single-stage surgery was performed in the other four cases; preoperative percutaneous biliary drainage was performed in three of them. The interval from diagnosis to definite surgery varied from case to case. Almost all of the cases managed by two-stage surgery might have been treated by single-stage surgery with preoperative biliary drainage, if they had no manifestation of diffuse peritonitis. Although it is difficult to clearly define the criteria for single-stage surgery for perforated CC, the lack of clinical signs of diffuse peritonitis or malignancy at initial assessment might be helpful factors for planning this elective surgery. It is controversial whether to plan single-stage or two-stage surgery. However, if the clinical outcomes for both strategies are the same, single-stage surgery would be better because it is potentially more cost-effective and safe. In addition to the two advantages described above, elective surgery when the patient’s condition has improved can have better results and avoid a possible emergency operation.

**Table 1:** Literature review for spontaneous perforated choledochal cyst in adults

Case no. [ref.]	Age (years) /sex	Nationality	Clinical presentation	Preoperative abdominal paracentesis	Choledochal cyst (Todani’s classification)	Preoperative drainage	Approach for drainage	Surgery (single- or two-stage)	The first surgical procedure	The second surgical procedure	Interval period from diagnosis to definitive surgery	Postoperative complications
1 [1]	28/F	Korea	Bile peritonitis	(+)	N/D	(−)	N/A	Single	Cyst excision	N/A	N/D	N/D
									Hepaticojejunostomy			
2 [5]	N/D	India	Bile peritonitis	(+)	Type I or type IVa	(−)	N/A	Two	T-tube choledochocystostomy	Cyst excision	4–8 weeks	N/D
										Hepaticojejunostomy		
									Peritoneal lavage			
3 [5]	N/D	India	Bile peritonitis	(+)	Type I or type IVa	(−)	N/A	Two	T-tube choledochocystostomy	Cyst excision	4–8 weeks	N/D
										Hepaticojejunostomy		
									Peritoneal lavage			
4 [5]	N/D	India	Localized biloma	(+)	Type I or type IVa	(+)	Percutaneous	Single	Cyst excision	N/A	4–8 weeks	N/D
									Hepaticojejunostomy			
5 [6]	24/M	Greece	Bile peritonitis	(−)	Type IVa	(−)	N/A	Two	T-tube choledochocystostomy	Cyst excision	N/D	N/D
										Hepaticojejunostomy		
									Peritoneal lavage			
6 [7]	25/M	Greece	Bile peritonitis	(−)	Type IVa	(−)	N/A	Two	T-tube choledochocystostomy	Cyst excision	8–12 weeks	SSI
										Hepaticojejunostomy		
									Peritoneal lavage			
7 [26]	24/F	Korea	Bile peritonitis	(−)	Type I	(+)	Percutaneous	Single	Cyst excision	N/A	1 week	None
									Hepaticojejunostomy			
8 [8]	28/F	India	Bile peritonitis	(+)	Type I	(−)	N/A	Two	T-tube choledochocystostomy	Cyst excision	12 weeks	N/D
										Hepaticojejunostomy		
									Peritoneal lavage			
9 [9]	25/F	India	Bile peritonitis	N/D	N/D	(−)	N/A	Two	T-tube choledochocystostomy	Cyst excision	N/D	N/D
										Hepaticojejunostomy		
									Peritoneal lavage			
10 [27]	18/F	Canada	Bile peritonitis	(+)	Type I	(+)	Percutaneous (+sphincterotomy)	Single	Cyst excision	N/A	8 weeks	None
									Hepaticojejunostomy			
Present case	28/F	Japan	Bile peritonitis	(−)	Type IVa	(+)	Endoscopic	Single	SSPPD	N/A	3 weeks	None

*F* female, *M* male, *N*/*A* not applicable, *N*/*D* not described, *SSI* surgical site infection, *SSPPD* subtotal stomach preserving pancreatoduodenectomy

## Conclusions

We experienced a successfully managed case of perforated CC in an adult who underwent endoscopic biliary drainage followed by a definitive single-stage surgery. This strategy has potential benefits in the management of patients without diffuse peritonitis or evidence of malignancy. It is difficult, but important, to determine proper timing of the definitive surgery. Long-term follow-up is also necessary because postoperative neoplastic change has been reported [36, 37].

## Consent

Written informed consent was obtained from the patient for publication of this case report and any accompanying images. A copy of the written consent is available for review by the Editor-in-Chief of this journal.

## Abbreviations

APBDU: anomalous pancreaticobiliary ductal union
CBD: common bile duct
CC: choledochal cyst
CT: computed tomography
EBS: endoscopic biliary stenting
ENBD: endoscopic nasal biliary drainage
ERCP: endoscopic retrograde cholangiopancreatography
